# One Health approach to controlling a Q fever outbreak on an Australian goat farm

**DOI:** 10.1017/S0950268815002368

**Published:** 2015-10-23

**Authors:** K. A. BOND, G. VINCENT, C. R. WILKS, L. FRANKLIN, B. SUTTON, J. STENOS, R. COWAN, K. LIM, E. ATHAN, O. HARRIS, L. MACFARLANE-BERRY, Y. SEGAL, S. M. FIRESTONE

**Affiliations:** 1 Communicable Disease Prevention and Control, Department of Health, Victoria, Australia; 2 Department of Infectious Diseases, Austin Health, Australia; 3 The Australian Rickettsial Reference Laboratory, Australia; 4 Asia-Pacific Centre for Animal Health, Faculty of Veterinary and Agricultural Sciences, The University of Melbourne, Australia; 5 Communicable Diseases Epidemiology and Surveillance, Department of Health, Victoria, Australia; 6 Department of Infectious Diseases, Barwon Health, Australia; 7 St John of God Pathcare, Geelong, Australia; 8 School of Medicine, Deakin University, Australia; 9 Department of Medicine, University of Melbourne, Australia; 10 Office of the Chief Veterinary Officer, Department of Environment and Primary Industries, Victoria, Australia

**Keywords:** Public health emerging infections, Q fever

## Abstract

A recent outbreak of Q fever was linked to an intensive goat and sheep dairy farm in Victoria, Australia, 2012-2014. Seventeen employees and one family member were confirmed with Q fever over a 28-month period, including two culture-positive cases. The outbreak investigation and management involved a One Health approach with representation from human, animal, environmental and public health. Seroprevalence in non-pregnant milking goats was 15% [95% confidence interval (CI) 7–27]; active infection was confirmed by positive quantitative PCR on several animal specimens. Genotyping of *Coxiella burnetii* DNA obtained from goat and human specimens was identical by two typing methods. A number of farming practices probably contributed to the outbreak, with similar precipitating factors to the Netherlands outbreak, 2007-2012. Compared to workers in a high-efficiency particulate arrestance (HEPA) filtered factory, administrative staff in an unfiltered adjoining office and those regularly handling goats and kids had 5·49 (95% CI 1·29–23·4) and 5·65 (95% CI 1·09–29·3) times the risk of infection, respectively; suggesting factory workers were protected from windborne spread of organisms. Reduction in the incidence of human cases was achieved through an intensive human vaccination programme plus environmental and biosecurity interventions. Subsequent non-occupational acquisition of Q fever in the spouse of an employee, indicates that infection remains endemic in the goat herd, and remains a challenge to manage without source control.

## INTRODUCTION

Q fever, a zoonosis caused by the small intracellular bacterium *Coxiella burnetii,* was first recognized clinically in 1935 [1]. Labelled ‘Query fever’ by Derrick; Burnet [2] and Cox [3] then characterized the causative agent, *C. burnetii,* which is endemic at varying prevalence on all continents except New Zealand and Antarctica [4, 5].

*C. burnetii* has been isolated from a wide range of mammals, birds and arthropods [6], including native animals [7–11]. In Australia, human infection is usually acquired from ruminants and is characterized by a non-specific febrile illness in about 40% of those infected [4]. Pneumonia, hepatitis or more rarely haematological, neurological or cardiovascular involvement can occur [12]. Morbidity increases with inoculation dose [13], and more cases are reported in males aged between 40 and 60 years [4, 12, 14]. A large outbreak in The Netherlands, 2007-2012, was attributed to intensive goat farming [14].

Within Australia, Q fever is known to be endemic in livestock in Queensland and New South Wales [15, 16]. These two states have some of the highest rates of notified human Q fever in the world with 50-110 cases/100 000 population per year [17]. Victoria has much lower rates (0·51 cases/100 000 for the last 10 years) than the national average (1·9 cases/100 000) (National Notifiable Diseases Surveillance System, Australian Government). Outbreaks in Australia are generally occupationally related, involving abattoir or rendering processes [18, 19], farmers [20], saleyards [21] and veterinary clinics [22, 23]. In Queensland in 2003, five cases were linked to a goat farm [24].

The mainstay in the prevention and control of outbreaks in Australia is vaccination of humans. The Q fever vaccine, (Q-Vax^®^, CSL Ltd, Australia), has been licensed since 1989 for use in adults. An Australian government-funded national vaccination programme for abattoir workers and farmers ran from 2001 to 2006; however, it did not target goat farmers [17]. Vaccine protection has not been evaluated in a randomized clinical trial; however, retrospective cohort studies estimate efficacy at >90% for those vaccinated 15 days prior to exposure [25]. Vaccination is strongly recommended for all occupational groups exposed to animals and their products [26].

We describe an outbreak that occurred on a 1450-ha commercial dairy goat and sheep farm in Victoria. Dairy sheep operations commenced in 1991 and dairy goat operations in 1995. The farm houses 5000 goats, 3000 of which are milking at any one time. The dairy sheep herd consists of ~2500 animals (~800 milkers), managed separately at pasture on an adjoining property. Processing of milk for retail products occurs in a high-efficiency particulate arrestance (HEPA) filtered factory. Goats are housed in open-sided sheds with deep straw bedding rather than at pasture; kidding occurs four times per year. The kids are removed from their mothers soon after birth (‘snatch reared’) and hand-fed to control for caprine arthritis encephalitis virus transmission. The owner reported that the number of abortions in the herd began to substantially increase from 2004 (detailed records not kept).

This paper describes the Q fever outbreak, the link to intensive goat farming and the One Health management approach.

## METHODS

### Epidemiological investigation

A Q fever outbreak investigation was launched by the Victorian Department of Health (DoH) on 11 February 2013 after the laboratory notification of five cases employed at the same farm within the week commencing 31 January 2013.

The DoH led the formation of a multi-disciplinary team tasked with investigating and managing the outbreak. This included members from the Victorian Department of Environment and Primary Industries (DEPI), The University of Melbourne (UoM), the Victorian regulatory body for workplace occupational health and safety (WorkSafe), the Australian Rickettsial Reference Laboratory (ARRL), St John of God Pathcare Geelong (SJOG), the Geelong Hospital Infectious Diseases Department (GH) and local General Practitioners (GPs). Two combined visits to the exposure site were undertaken for the purposes of risk assessment by DoH, DEPI, UoM, SJOG and ARRL, with a series of smaller focused follow-up visits.

In Australia, Q fever is a notifiable disease in humans but not animals [27, 28]. In Victoria, notification of human cases must be made in writing by clinicians and diagnostic laboratories. Public health officers follow-up all notifications, undertaking a structured questionnaire-based interview with the treating doctor and case. The aim is to identify the source of acquisition, record clinical details and to instigate appropriate public health interventions.

A confirmed human case of Q fever requires either definitive laboratory evidence (detection of *C. burnetii* by nucleic acid testing or by culture, seroconversion or a significant increase in antibody level to Phase II antigen in paired sera tested in parallel in the absence of recent Q fever vaccination); or a clinically compatible syndrome accompanied by detection of IgM specific for Phase II *C. burnetii* in the absence of recent Q fever vaccination [29]. A probable human case was defined as a person with an epidemiological link to the outbreak and either a clinically compatible illness or laboratory evidence of recent infection [total Phase II indirect immunofluorescence assay (IFA) ⩾200].

The index case was a 61-year-old male (case 11), who presented to his GP on 21 January 2013 with fever, headache, sweats and malaise. The GP noted his occupation on a goat farm and the burial of goat carcasses in the previous month. Q fever was confirmed by a positive specific IgM enzyme immunoassay. Four of his co-workers (cases 2, 3, 9, P1) with similar illnesses subsequently requested testing (Table 1).

**Table 1.** Cases of acute Q fever during an outbreak of Q fever in Victoria, 2012–2014

Case no.	Symptom onset date	Sex	Age, years	Laboratory diagnosis	Total IFA titre Ph2	Sought care	Hospitalized	Treated	Vaccinated	Role on property
1	1 Mar. 2012	F	39	Specific IgM	3200	GP, ED	Y	Y	N	Electrician
2	20 Aug. 2012	M	60	Specific IgM	>3200	N	N	N	N	Tradesperson
3	1 Sept. 2012	F	40	Specific IgM	>3200	N	N	N	N	Administration
4	22 Sept. 2012	M	30	Specific IgM	>3200	N	N	N	N	Milking, kidding
5	27 Sept. 2012	F	25	Specific IgM	>3200	GP	N	N	N	Milking, kidding
6	7 Dec. 2012	M	40	Specific IgM	>3200	N	N	N	N	Farm labourer
7	9 Dec. 2012	M	41	Specific IgM	200	N	N	N	N	Process manager#
8	10 Dec. 2012	F	48	Specific IgM	>3200	GP	N	N	N	Administration
9	21 Dec. 2012	M	51	Specific IgM	>3200	N	N	N	N	Mechanic
10	22 Dec. 2012	F	53	Specific IgM	1600	N	N	N	N	Administration
11	12 Jan. 2013	M	61	Specific IgM	EIA positive	GP||	N	Y	N	Farm labourer
12	13 Jan. 2013	M	24	Specific IgM	>3200	N	N	N	N	Pasteurization#
13	12 Sept. 2013	M	36	Culture†	n.a.	ED||	N	Y	N	Milking
14	13 Sept. 2013	F	36	Seroconversion†‡	n.a.	GP, ED||	Y	Y	N	Milking, kidding
15	15 Oct. 2013	M	60	Seroconversion	n.a.	ED||	Y	Y	N	Farm labourer
16	16 Oct. 2013	F	17	Rising titre‡	800	N	N	N	N	Bottle-feeding kids
17	26 Oct. 2013	M	29	Culture†	n.a.	GP, ED||	Y	Y	25 Oct. 2013	Carpenter
18*	4 July 2014	F	55	Seroconversion	n.a.	GP, ED||	Y	Y	N	n.a.
**Probable cases**										
P1	Asymptomatic	M	22	Specific IgM	1600	N	N	N	N	Milking
P2	Asymptomatic	F	48	Specific IgM	>3200	N	N	N	N	Administration
P3	Asymptomatic	F	33	Specific IgM	200	N	N	N	N	Milking
P4*	Asymptomatic	M	86	Specific IgM	>3200	N	N	N	N	Milking, kidding
P5*	Asymptomatic	F	69	Specific IgM	800	N	N	N	N	Farm labourer
P6*	13 Sept. 2013	M	14	n.a.§	EIA negative	ED||	N	Y	N	Milking

IFA total phase 2 titre was designated not applicable (n.a.) when the diagnosis was confirmed by culture or seroconversion, as the total titre response is not relevant for meeting the case definition. ‘Sought care’ indicates sought care at time of illness via a General Practitioner (GP), Emergency Department (ED) or neither (N).

* Not an employee, see text.

† PCR undertaken, negative result.

‡ Culture attempted, nil isolated.

§ Repeat serology not undertaken.

|| QF diagnosis made at time presented to healthcare.

# Tasted unpasteurized milk.

On 11 February 2013 the farm was advised of a formal outbreak investigation and was instructed to institute active workplace surveillance. Skin and serological screening was subsequently undertaken for employees on 19 February 2013 by a doctor attending the workplace. DEPI launched a Significant Disease Investigation, including risk assessment, management recommendations and livestock testing.

Data on potential risk factors for all of the farm's employees during 2012-2014 were collated in Microsoft Excel and analysed as a cohort study using Stata version 13.0 (Stata Corporation, USA). Descriptive analysis was undertaken with frequency tables by case status and calculation of crude attack rates (based on incident confirmed and probable outbreak cases), excluding those employees with positive skin or serology tests at screening. Those variables with univariable associations (applying a liberal *P* < 0·20 cut-off) with case status were entered into a multivariable generalized linear model with robust standard errors according to Zou [30], and retained if associated with case status at *P* < 0·05. Age and sex were considered *a prior* as confounders and forced into the final model.

### Livestock and environmental investigation

Goats from the largest herd were sampled for serology on 8 August 2013. This herd comprises about 1200 milking animals. A single bleed of 65 goats, randomly selected from two non-pregnant milking herds of about 400 goats, was undertaken to demonstrate the presence of disease with 95% confidence at a design prevalence of 5%, adjusting for finite population size [31].

During subsequent kidding seasons, samples were obtained for quantitative polymerase chain reaction (qPCR) testing including aborted placentas and fetuses, vaginal swabs from associated does, and environmental samples (from bedding and air testing).

### Laboratory investigation

Human samples were tested at ARRL. Serological testing was performed using a nationally accredited (National Association of Testing Authorities, Australia) in-house IFA, in which a fourfold rise in antibody titres between acute and convalescent sera, or the presence of antibodies to Phase II IgM antigen only was considered a positive result. Patients’ sera were screened at dilutions of 1/25 and 1/400, the latter to detect the prozone phenomenon. If positive the antibody titre was determined by titration. Samples were selected for qPCR from cases that were identified as being part of the outbreak at illness onset; had suitable specimens available (acute serum or EDTA blood); and were Phase II IgM negative on their initial serology test [32]. DNA was extracted from the serum or buffy coat fraction using a commercial kit (RBC Bioscience, Taiwan). Assays targeting the *C. burnetii com1* gene and the heat shock operon htpAB (primers htpAB_F: 5’-GTGGCTTCGCGTACATCAGA-3’ and htpAB_R: 5’-CATGGGGTTCATTCCAGCA-3’, and probe htpAB_P: 5’-FAM-AGCCAGTACGGTCGCTGTTGTGGT-BHQ1-3’), were performed [33], with the result considered positive if both targets were detected or a single target detected in duplicate reactions. Isolation of *C. burnetii* was attempted from four of the acute serum samples by inoculation of the serum into a Vero cell culture, monitored for up to 12 weeks [34].

Goat specimens and environmental samples were tested using the *com1* qPCR based on collection and extraction methods described previously [35, 36]. Culture was attempted from two of the highly positive samples, with swabbed material resuspended in PBS filtered sequentially through a 0·45 µm and 0·22 µm filter. The filtrates were added to Vero cell monolayers and cultures maintained as above.

Two genotyping methods based on single nucleotide polymorphisms [37] and the insertion element IS*1111* [34] were applied to the isolates and a selection of the PCR-positive samples.

The goat sera were tested at DEPI by the complement fixation test (CFT) using *C. burnetii* Phase II antigen (Siemens Healthcare Diagnostics, Germany). Any sample with a CFT titre of ⩾8 was retested using *C. burnetii* and control antigen; inconclusive results and non-specific reactors were eliminated from further analyses.

### Ethical standards

The authors assert that all procedures contributing to this work comply with the ethical standards of the relevant national and institutional guides on the care and use of animals. All animal sampling was undertaken under the Victorian Government Department of Environment and Primary Industries’ significant disease investigation programme.

## RESULTS

### Epidemiological investigation

**Fig. 1.**
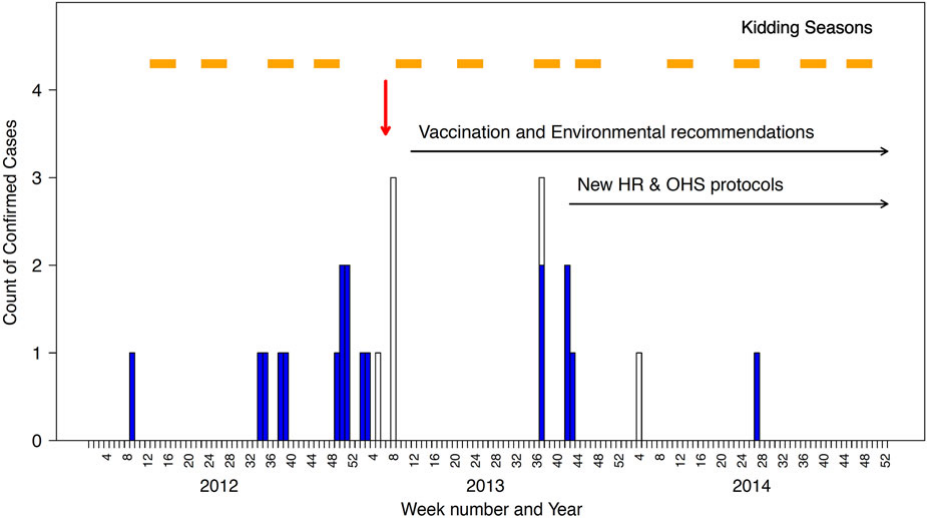
Epidemic curve of confirmed cases of Q fever linked to an outbreak associated with a goat and sheep dairy farm in Victoria, 2012–2014. Light blue bars represent confirmed cases, open bars are probable cases and the red arrow indicates when the investigation was launched.

By 28 February 2013, 12 cases of acute Q fever had been confirmed with symptom onset between 1 March 2012 and 31 January 2013 (Table 1, Fig. 1). A series of public health measures designed to curtail the outbreak were implemented as described in ‘public health actions’ (see Table 2). Despite these measures, a further five workers were confirmed with acute Q fever during the September kidding season. Four of these workers had not received their recommended vaccination, while the fifth (case 17) became unwell the day after vaccination. Case 17 improved when administered doxycycline, and with case 13, was confirmed as an acute case after *C. burnetii* was cultured from blood.

**Table 2.** Public health actions

General biosecurity measures	Case-finding and management
• Biosecurity hazard signage erected on all the public access entrances to the property • All fences and gates were to be maintained in good working order with access gates locked if possible • Vehicle wash stations and footbaths • Hand wash or disinfection facilities	• Active surveillance and screening • Diagnosis of Q fever discussed with the cases involved, follow-up organized with an Infectious Diseases specialist • Letters sent to GPs in the region recommending a heightened awareness, and index of suspicion, encouraging testing and details on how to notify any suspected cases • Chief Health Officer Advisory release to medical practitioners in the region, detailing the increased incidence of Q fever
Employee and visitor risk reduction	Environmental and animal management
• Vaccination strongly recommended for all employees and visiting contractors, with documentation to be sighted and recorded • Coordinated screening and vaccination process for new staff developed in conjunction with a local GP, with records forwarded to the National Q Fever Registry ○ Vaccination required 15 days prior to visiting the property ○ If this not possible then personal protective equipment (PPE) to be utilized in the interim • All visitors to the property required education on risk, both oral and written ○ Regular visitors required to show evidence of vaccination, facilitated by the establishment of a visitor registry ○ Irregular visitors provided with PPE • Information and policy regarding Q fever incorporated into occupational health and safety policies and procedures for the business, including the orientation package for new employees	• Manure should not be removed from the deep litter sheds or yards for at least 1 month after the kidding season, then should be composted or alternatively stored for 3 months prior to spreading on farm land for fertilizer • Manure required to be covered during storage and transport and must be under-ploughed immediately when spreading on farming land • All abortive materials should be removed immediately and safely disposed of by deep burial or composting • Kids not allowed to be sold younger than 2 month of age, and all prospective buyers of older goats must be notified of the Q fever status of the farm

Nine months later (4 July 2014), a 55-year-old female (case 18) presented to her GP with symptoms of headache, chills and significant sweats. She was subsequently hospitalized and Q fever confirmed by seroconversion. As the spouse of case 15, she was not employed on the goat farm and had not been on or near the property in 2014. She visited farmhouses on other sheep properties in the district, but did not have any direct animal contact. Fomite transmission (washing her husband's contaminated clothing) was thought to be the most likely route of infection. Sexual transmission is a possibility, but considered less likely having been much less frequently described, with no cases documented following antimicrobial treatment of the index case [4, 38].

A total of 18 confirmed cases were identified (10 males, 8 females), with a median age of 40 years (interquartile range 30–53; Table 1). None of the cases were appropriately vaccinated. Two cases were involved in tasting unpasteurized goat's milk, in addition to other exposures. Nine of the 18 cases sought healthcare at the time of their illness, of which four cases were hospitalized with a severe influenza-like illness. These four cases were diagnosed with acute hepatitis (alanine transaminase peak 3–15 times the upper limit of normal reference range); two patients also suffered moderate thrombocytopenia. No case required intensive care support. One case was hospitalized after acute illness for investigation of a chronic fatigue-like syndrome. The remainder of the cases suffered from either a mild or moderate influenza-like illness. One case became pregnant 5 months after her untreated acute Q fever infection (case 3) and subsequently delivered a healthy infant at term. Overall, only seven of the 18 cases received specific treatment for Q fever (doxycyline).

In addition to the 18 confirmed cases, there were six probable cases, including five asymptomatic cases with high Phase II titres and one clinically ill case without confirmatory serology (Table 1).

Eighteen further employees had low positive screening results (IFA total antibody <200 or positive skin test). Given they all lived and worked in a rural area, it was not possible to conclude whether these reflect individuals with asymptomatic infection or exposure to *C. burnetii* separate to this outbreak (see Supplementary Table S1 for further screening results).

**Fig. 2.**
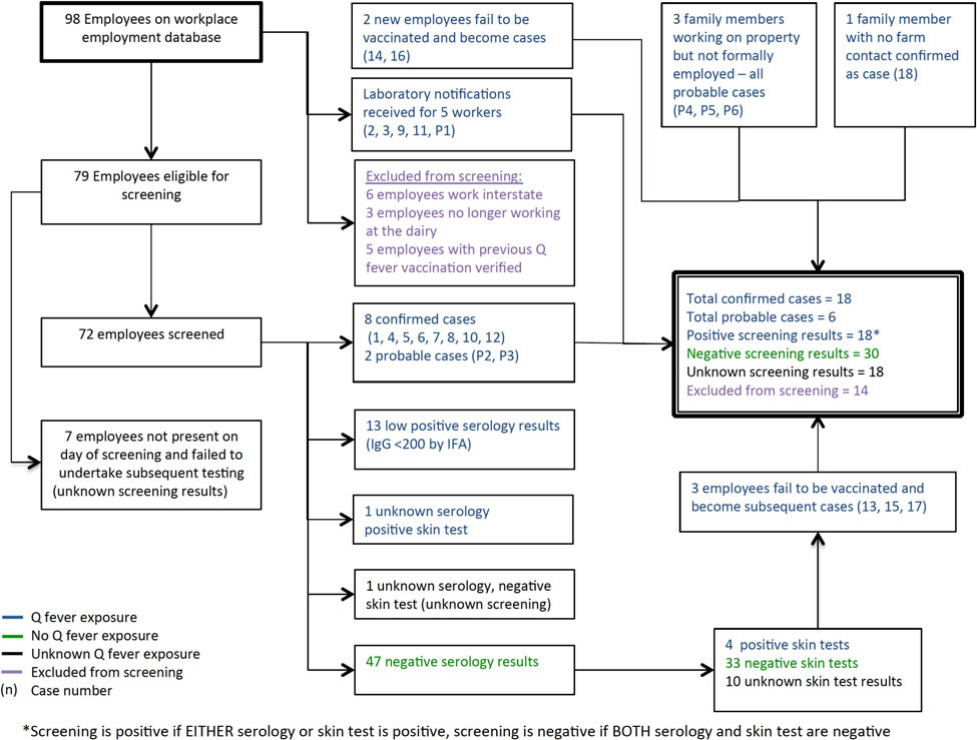
Relationship between cases, probable cases and employees screened for Q fever.

Of 100 farm employees, five had been vaccinated prior to detection of the outbreak and six were employed in marketing roles with no contact with the affected farm; these were excluded from further analyses (Fig. 2). The case status and details of the remaining 89 employees are presented in Table 3. Eighteen employees had positive screening results, leaving a crude attack rate of 31% (22 confirmed and probable cases in 71 employees). The highest attack rates were observed in administrative workers in the office adjacent to the main dairy shed (75% female, none vaccinated) and persons aged >40 years; while the lowest rate was seen for those working in the HEPA-filtered factory. Adjusting for age and sex, administrative workers and those regularly handling goats and kids had >5 times the risk of infection of workers in the HEPA-filtered factory (Table 4).

**Table 3.** Risk factors for Q fever infection in employees of a goat dairy farm, Victoria, 2012–2014

		Confirmed cases								
Variable (*n*)	Non-cases	Pre-screening	Post-screening	Probable cases	Screening positives	Incident cases*	At risk†	AR	RR	95% CI
Job description‡										
Administration (8)	3	3	0	1	1	4	7	0·57	7·43	1·68–32·9
Goat milker (17)	7	2	2	2	4	6	13	0·46	6·00	1·39–26·0
Farm labourer (16)	9	5	1	0	1	6	15	0·40	5·20	1·18–22·8
Goat and kid handling (7)	4	0	2	0	1	2	6	0·33	4·33	0·75–25·2
Factory worker (36)	24	2	0	0	10	2	26	0·08	1·00	—
Unknown (5)										
Site of work‡										
Office (8)	3	3	0	1	1	4	7	0·57	5·14	1·47–18·0
Dairy and goat sheds (25)	12	2	4	2	5	8	20	0·40	3·60	1·08–12·0
Farm-wide (14)	8	4	1	0	1	5	13	0·38	3·46	0·96–12·4
Milk/cheese factory (37)	24	3	0	0	10	3	27	0·11	1·00	—
Unknown (5)										
Sex										
Male (42)	21	7	3	1	10	11	32	0·34	1·49	0·70–3·16
Female (47)	30	5	2	2	8	9	39	0·23	1·00	—
Age, years§										
10–19 (9)	6	0	1	0	2	1	7	0·14	1·00	—
20–29 (22)	13	2	1	1	5	4	17	0·24	1·65	0·22–12·4
30–39 (18)	12	1	2	1	2	4	16	0·25	1·75	0·23–13·2
40–49 (18)	4	7	0	1	6	8	12	0·67	4·67	0·72–30·4
50–59 (13)	10	0	0	0	3	0	10	0·00	Undef.	—
⩾60 (5)	2	2	1	0	0	3	5	0·60	4·20	0·59–30·0
Unknown (4)										

AR, Crude (unadjusted) attack rate; RR, unadjusted relative risks; CI, confidence interval, estimated with univariable regression models.

* Confirmed and probable cases only.

† At risk excludes those with positive screening results as it is unknown when they seroconverted.

‡ Data missing for five employees

§ Data missing for four employees.

**Table 4.** Final model of risk factors for Q fever infection in employees of a goat dairy farm, Victoria, 2012–2014

Variable	Coeff.	s.e. (Coeff.)	Adjusted RR	95% CI
Job description				
Administration	1·70	0·74	5·49	1·29–23·4
Goat milker	1·73	0·84	3·89	0·71–21·3
Farm labourer	1·36	0·87	4·01	0·70–22·9
Goat and kid handling	1·39	0·89	5·65	1·09–29·3
Factory worker	0·00	Ref.	1·00	—
Sex				
Male	0·11	0·48	1·25	0·44–2·86
Female	0·00	Ref.	1·00	—
Age, years*				
⩾40	0·37	0·43	1·88	0·62–3·33
10–39	0·00	Ref.	1·00	—

*n* = 63; log-likelihood = −38·6473; df = 7; Akaike's Information Criterion (AIC) = 91·29.

Coeff., Coefficient; s.e., standard error; RR, relative risks (adjusted for other variables in the model); CI, confidence interval.

* Collapsed to binary variable for numerical reasons.

### Livestock and environmental investigation

Nine of the 65 goats sampled had CFT antibody titres for *C. burnetii* (apparent prevalence = 15%, 95% confidence interval 7–27); six had uninterpretable results. Age could not be ascertained for one test-negative animal. qPCR results of samples from goat vaginal swabs, placenta and aborted kids are presented in Table 5. None of the air or bedding samples conclusively tested positive.

**Table 5.** Serological and quantitative polymerase chain reaction (qPCR) assay results from goat and environmental samples collected during an outbreak of Q fever in Victoria, 2013–2014

									
Complement fixation test titres (IgM or IgG, *C. burnetii* Phase II specific)					qPCR assay for *C. burnetii*								
	Age, years							Cycle threshold (C_t_)					
Titre	<2	2–4	⩾5	Unknown	Sample type	No. of samples	Positive	<20	20–30	30–35	>35	Inconc.	Negative
8	0	1	1	0	Goat vaginal swab	15	4	2	1	1	−		11
16	3	2	0	0	Placenta	9	6	3	−	1	2	−	3
32	0	1	0	0	Aborted kids	22	18	3*	5	6	4	−	4
64	0	0	0	0	Live kid	2	−	−	−	−	−	−	2
128	0	1	0	0	Air sample (500 l)	3	−	−	−	−	−	1	2
Negative	28	17	4	1	Goat faeces	1	−	−	−	−	−	−	1
Inconc.	3	3	0	0	Bedding	3	−	−	−	−	−	1	2
Apparent prevalence	9·7%	22·7%	20·0%	–									

* One sample, C_t_ = 11·94, culture obtained from this specimen.

Inconc., Inconclusive results. For qPCR, only one target present or a single target present in only one of duplicate reactions.

A pure culture of *C. burnetii* was isolated from a stillborn kid, and was genotyped along with the two human isolates and eight of the strongly positive qPCR samples. Identical single nucleotide polymorphism patterns were observed in all outbreak isolates and samples, identifying a multispacer sequence typing (MST) genotype that has been observed in other Australian isolates but is novel compared to previously published data [34]. This novel MST genotype is most closely related to genotypes MST1–MST7 observed in animal and human isolates from France, Ukraine, Russia and Kyrgyzstan, and to MST30 observed in the Namibia strain isolated from an aborting goat, but genetically distinct from the Netherlands epidemic genotype MST33 [39]. Strains were also identical by the IS*1111* typing method.

Risk factors contributing to environmental contamination included: high density of goats in close proximity (~15 m) to offices; multiple kidding seasons per year; dead animals buried in pits on site; straw and manure from the sheds spread directly onto pasture without composting [5]; failure to routinely wash or disinfect vehicles travelling between farm sites; and below average rainfall in the 6 months prior to the index case (Australian Bureau of Meteorology).

### Public health actions undertaken

Following the risk assessment, the outbreak team developed a series of recommendations initially targeting those at risk due to exposure to infectious organisms on the property, and then extended to reduce exposure of persons in the region surrounding the property (Table 2).

Farm management are implementing an education campaign for family members, with optional vaccination through local GPs. Uniforms are to be introduced, which will be washed and laundered on the property; work boots will remain on site. There is a longer-term plan to develop showering and changing facilities for staff.

## DISCUSSION

We describe the largest Q fever outbreak related to farming in Australia, and the coordinated, multidisciplinary approach to its management. Epidemiological and molecular links established goats as the likely source. High rates of infection were observed in unvaccinated workers in regular close contract with goats and workers in the office next to the main dairy. Lower rates of infection in cheese factory workers suggests they were protected by the HEPA-filtered air, and therefore that wind-borne spread of infectious organisms may have significantly contributed to acquisition of infection in other worker groups. Animal and environmental investigations highlighted particular farming practices that contributed to endemicity within the goat herd and subsequent human exposure. Fomite transmission appears to have occurred late in the outbreak, indicating ongoing local environmental contamination.

The origin of infection in the goats at the centre of this outbreak has not been definitively established, *C. burnetii* may have entered the herd in 2011 when goats were introduced from interstate. This is an ongoing area of active research. Whereas *C. burnetii* infection is endemic in livestock and wildlife in other Australian states [7–11, 15], the prevalence of coxiellosis in livestock or wildlife in Victoria remains unknown. Traditionally, infections in animals in Victoria have been attributed to exposure to livestock introduced from other states.

The goat farm at the centre of this outbreak utilizes an intensive system for breeding and milking goats uncommon in Australia, but resembling that of goat farms involved in the recent Netherlands outbreak [40]. This system provides an ideal environment for the multiplication, persistence and spread of *C. burnetii* infection in goats, with potential for infection of farm workers and the sheep flock. Contributing factors include: high stock density facilitating direct animal-to-animal spread; housing on deep straw bedding allowing build-up of a high level of infectious organisms and use of discarded bedding as manure for the paddocks thus introducing the bacteria to animals at pasture. Multiple kidding seasons increases the frequency of infectious shedding, environmental contamination and high-risk periods for transmission to humans. It also repeatedly introduces large number of susceptible young goats to the contaminated environment. Each of these factors in concert may aid in the establishment and maintenance of Q fever endemicity.

In common with the Netherlands outbreak [5, 14], the early involvement of a multi-disciplinary team allowed a comprehensive risk evaluation and consensus control recommendations. Further research needs were identified, including a review of the epidemiology of *C. burnetii* infections in humans in Victoria, and validation of the IFA for use in establishing the prevalence in Australian livestock and wildlife species.

There are marked similarities between the setting of this and the Netherlands outbreak. Both occurred in a region with previously low Q fever notification rates [14] in therefore highly susceptible human and livestock populations, and on farms with high goat densities [14]. Slow recognition of both outbreaks led to a delay in instigating control measures [41].

Obvious differences include the vastly lower numbers of human infections involved in the Australian outbreak, predominantly occupational acquisition of infection and, as yet, no clear evidence of dissemination to sheep and goat farms outside this enterprise. The considerably lower human and livestock population density surrounding the Australian goat farm has presumably contributed to many of these differences. Epidemiological studies in The Netherlands found the greatest infection risk for community members was within 2 km, with minimal risk beyond 5 km [42]. The closest small town to the Australian farm is >10 km away and has <1000 residents (Australian Bureau of Statistics, 2011 Census data). Employees predominantly occupy farmhouses closest to the property. There are no other intensive livestock farms nearby, only those with more typical extensive pasture-based farm systems. Due to the much smaller scale of this outbreak and different risk profile for surrounding human populations, a number of control measures instigated in The Netherlands were considered but not adopted in the control of this Australian outbreak (Table 6).

**Table 6.** Features relating to this outbreak as compared to The Netherlands Q fever outbreak

	This outbreak	The Netherlands [12]
	Occupational exposure	Community exposure
**Outbreak features**		
Cases		
Low population prevalence prior to outbreak	✓	✓
Delay in recognition of outbreak	✓	✓
Spread of infection to other farms	×	✓
Risk factors		
Intensive goat farming	✓	✓
Close proximity to densely populated areas	×	✓
Windborne spread of disease	×*	✓
**Public health management**		
Screening of population	✓	×
Animal infections notifiable	×	✓
Vaccination of humans	✓	×†
Vaccination of animals	×‡	✓
Manure and contaminated materials management	✓	✓
Bulk milk testing	×	✓
Restriction on breeding	×	✓
Restriction on transport	×	✓
Culling of animals	×	✓

* Only locally to others on property.

† Recent use in high risk populations [40].

‡ Application for importation permit rejected, further research ongoing to develop vaccine suitable for use in Australian livestock.

Another clear difference is the availability of animal *vs.* human vaccines. While human vaccination appears to have been the most effective short-term intervention, it has not addressed the challenging issue of infection in the goats. Control at source has therefore not been achieved. Human and livestock vaccination have their own advantages and provide complementary infection control benefits. The human vaccine is of direct benefit to those with inescapable occupational exposure to infectious organisms or those with background medical conditions that increase complication risk. However up-to-date vaccination of all staff members has proven difficult to maintain in this fluctuating large workforce, and the final case highlights the risk in family members who are not the target of the vaccination intervention. While the livestock vaccine does not prevent infection [43], the reduction in excretion of infectious particles from vaccinated animals (and therefore reduction in environmental contamination) may significantly contribute to source control. For this reason, efforts were made to import the inactivated Phase I livestock vaccine (Coxevac^®^, CEVA Santé Animale, France). The import application was rejected by the Department of Agriculture due to biosecurity concerns, while currently the human vaccine Q-Vax (CSL Ltd) is prohibitively expensive for application in livestock (CSL, personal communication). The Netherlands Ministry of Agriculture granted a special dispensation for use of this same animal vaccine [44] and to import the human vaccine from Australia for particular at-risk groups [45]. Efforts are now underway to develop a vaccine locally that may be administered to goats on the farm as this is seen as the most feasible way of sustainably reducing the risk of human infections in the long term.

The key limitations of this study relate to the retrospective identification of many of the cases. This, combined with the non-specific clinical illness of Q fever, may have led to underreporting of cases. It is too early to comment on any of the long-term consequences suffered by the cases due to their infection. It also remains premature to conclude that the outbreak has been completely controlled, hence on-farm active human and animal surveillance continues.

## CONCLUSIONS

Intensive goat farming is a growing industry in Australia. Without the routine implementation of *Coxiella* preventative measures, the risk of human Q fever cases is likely to rise, despite clear lessons from the Dutch outbreak. This outbreak highlights the substantial challenges of preventing human illness in a setting where Q fever vaccination for animals is not available, and provides further evidence as to the ability for *C. burnetii* to thrive on intensively managed goat farms. In this Australian setting, human vaccination 15 days prior to exposure was instrumental in preventing further cases of acute Q fever. We therefore advocate for mandatory vaccination of all staff, in addition to routine implementation of *Coxiella* preventative measures on such farms. This outbreak illustrates the infectious risk management complexities in such an environment; the need to address the triad of human–animal–environmental aspects; and the essential requirement of a One Health approach in investigation and management.

## Supplementary material

For supplementary material accompanying this paper visit http://dx.doi.org/10.1017/S0950268815002368. click here to view supplementary material
