# Supplementary material for: One Health approach to controlling a Q fever outbreak on an Australian goat farm
Source: Epidemiol Infect. 2015 Oct 23;144(6):1129–41. doi: 10.1017/S0950268815002368 (PMC4825098; doi:10.1017/S0950268815002368)
Supplement: Supplementary file 1 [file S0950268815002368sup001.docx]

**SUPPLEMENTARY MATERIAL**

**Supplementary Table S1. *Serological and skin testing results for 56 employees***

|  | | **Serology Result** | |  |
| --- | --- | --- | --- | --- |
|  |  | **Positive** | **Negative** | **Total** |
| **Skin**  **testing result** | **Positive** | 15 | 4 | **19** |
|  | **Negative** | 2 | 32 | **34** |
|  | **Equivocal** | 2 | 1 | **3** |
|  | **Total** | **19** | **37** | **56** |

In total 72 employees presented for screening, of which 56 completed both serological and skin testing. 14 employees undertook serological testing but failed to return at one week for reading of their skin test result, of these 4 were serologically positive and therefore recorded as a positive screening result regardless of the skin test result, 10 were serologically negative and are therefore recorded as unknown. 2 employees had skin but not serological testing of which one was positive and one was negative. Employees with positive serology and equivocal skin test were recorded as positive for screening, the one employee with negative serology and equivocal skin test was recorded as negative for screening and was therefore vaccinated.

See Figure 2 for further details.
